# Interaction Between Microbiota and Immunity: Molecular Mechanisms, Biological Functions, Diseases, and New Therapeutic Opportunities

**DOI:** 10.1002/mco2.70265

**Published:** 2025-06-19

**Authors:** Jingjing Zeng, Zimeng He, Guoqing Wang, Yuxin Ma, Feng Zhang

**Affiliations:** ^1^ Key Laboratory of Basic Pharmacology of Ministry of Education and Joint International Research Laboratory of Ethnomedicine of Ministry of Education and Key Laboratory of Basic Pharmacology of Guizhou Province and Laboratory Animal Center Zunyi Medical University Zunyi Guizhou China

**Keywords:** microbiota, immunity, molecular mechanisms, biological functions, diseases, new therapeutic opportunities

## Abstract

The microbiota is pivotal for our health. It includes different phyla like Bacteroidetes, Firmicutes, Actinobacteria, Proteobacteria, Fusobacteria, and Verrucomicrobia. The interaction between microbiota and immunity shares a bidirectional relationship. The microbiota helps to stimulate immunity development. The immunity influences microbial composition in turn. This interaction is critical for maintaining homeostasis, preventing pathogen invasion, and regulating the immune system. Furthermore, this symbiotic relationship is crucial for maintaining overall health and preventing various diseases. The microbiota–immune system contributes to immune system maturation, while the immune system selects for beneficial microbiota composition, thus enhancing our immunity. This review summarizes the molecular mechanisms and biological functions of the interaction between microbiota and immunity, offering solid evidence for the role of microbiota in immune regulation. Notably, the review categorizes microbiota according to phyla and explains disease associations, molecular effectors, and functional outcomes about the microbiota–immune system. We also introduced three core molecular mechanisms of the microbiota–immune systems. Moreover, we detail the progression from target discovery to clinical trial design for bacterial and immune‐related diseases. Finally, we propose four therapeutic strategies for diseases.

## Graphical abstract

This review systematically concludes the interaction between microbiota and immunity. We introduce six phyla: Bacteroidetes, Firmicutes, Actinobacteria, Proteobacteria, Fusobacteria, and Verrucomicrobia. We also elucidate molecular mechanisms, biological functions, diseases, and new therapeutic opportunities between microbiota and immunity.

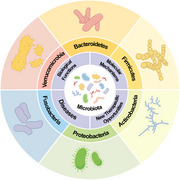

## 1 Introduction

The microbiota, comprised of bacteria, archaea, fungi, and viruses, is crucial for immunity [1]. It markedly influences immunity, with its composition linked to various diseases such as immunological diseases [2], neurodegenerative diseases [3], digestive diseases [4], and cardiovascular diseases [5]. Changes in microbiota have been shown to serve as potential biomarkers for many diseases [6]. The relation between microbiota and human health is increasingly recognized, with microbiota composition representing a vast array of cells and functions that can be viewed as an additional organ.

Immunological diseases cover a wide range of conditions. Dysbiosis is associated with several immunological diseases [7] such as rheumatoid arthritis (RA), type 1 diabetes (T1D), type 1 diabetes (T2D), multiple sclerosis (MS), acquired immune deficiency syndrome, systemic lupus erythematosus (SLE), allergic diseases (such as allergic rhinitis, atopic dermatitis, and food allergy), and inflammatory bowel disease (IBD), which include ulcerative colitis (UC), Crohn's disease, and colorectal cancer (CRC) [8]. The microbiota that initially colonized the gut essentially helps to train the developing immune networks, acting as essential teachers during this critical growth phase. Microbiota communicates with the innate and adaptive immune systems, influencing processes such as immune cell differentiation, cytokine production, and the maintenance of immunological tolerance [9]. The microbiota interacts closely with immune defenses, forming the most extensive frontline protection system. It plays a crucial role in maintaining the integrity of the barrier, preventing the translocation of pathogens and their products into systemic circulation. Beneficial compounds like short‐chain fatty acids (SCFAs) are created when gut microbes break down dietary fiber, actively regulate immune responses, and reduce inflammation [10]. However, when referring to dysbiosis, research increasingly links such imbalances to the progression of immune‐related disorders.

This review systematically covers research on the microbiota–immune system interactions over the past 3 years. Notably, it categorizes microbiota by phyla and explains their disease associations, molecular effectors, and functional outcomes within the microbiota–immune system. We also introduced three core molecular mechanisms of the microbiota–immune systems, namely, mucosal barrier dynamics, pattern recognition receptors (PRRs) signaling networks, and metabolite‐mediated epigenetic regulation. In addition, we elaborated on bacterial and immune‐related diseases from target discovery, organoid screening, animal validation, to clinical trial design. Interestingly, we provide four therapeutic strategies for diseases, including dietary interventions, biotics engineering, fecal microbiota transplantation (FMT) clinical paradigms, and pharmacomicrobiomics.

## 2 Core Mechanisms of Microbiota–Immune Crosstalk

The mechanisms of microbiota–immune crosstalk intrigued many research. Although the mechanisms of microbiota–immune system are intricate, three core mechanisms of microbiota–immune crosstalk stand out.

### 2.1 Barrier Dynamics

**FIGURE 1:**
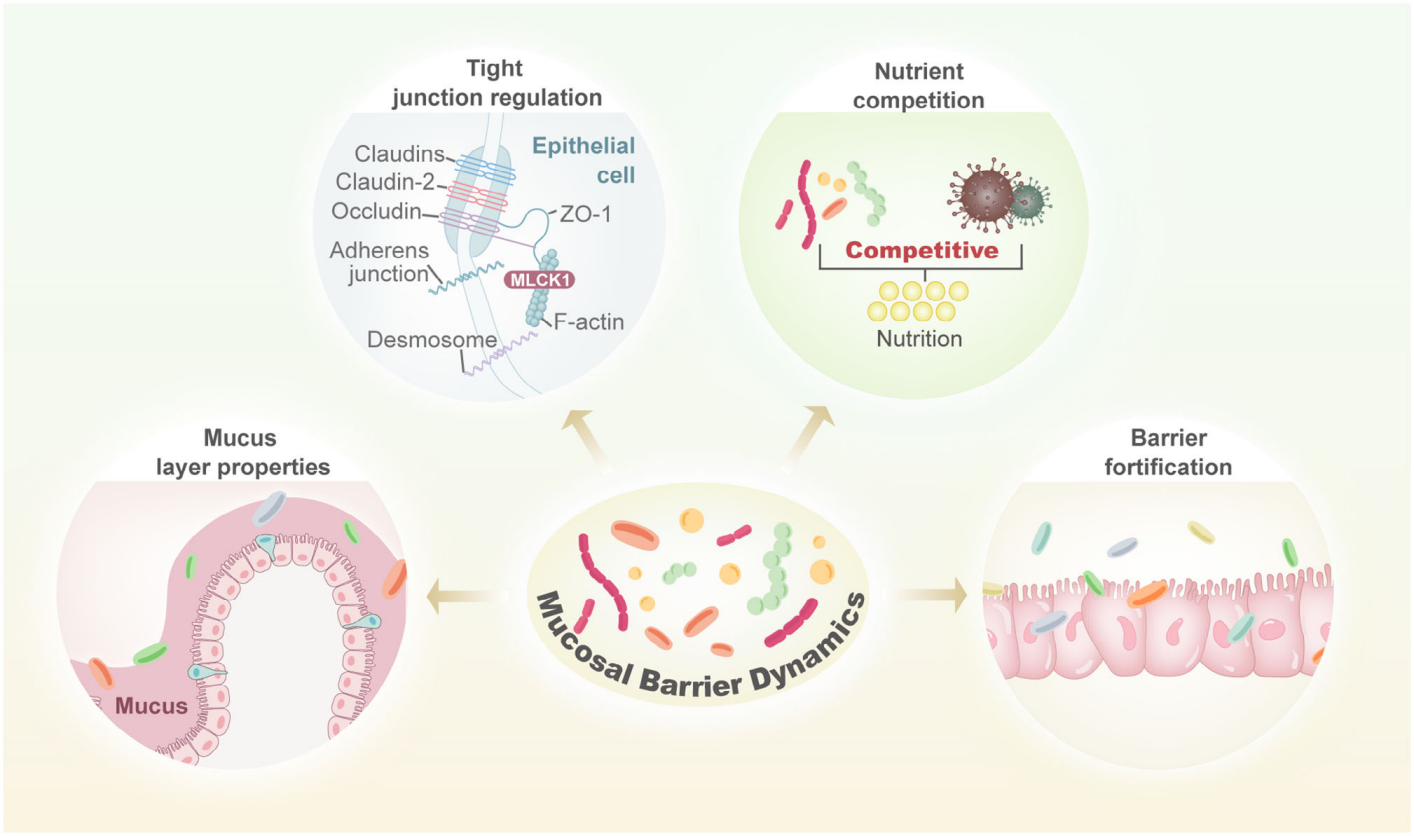
Mechanisms of microbiota in mucosal barrier dynamics. Mucosal barrier dynamics of microbiota can be divided into four parts, mucosal layer properties, tight junction regulation, nutrient competition, and barrier fortification.

As the largest symbiotic community with the host, the microbiota plays a crucial role in maintaining intestinal homeostasis. Imbalances of the microbiota, namely, dysbiosis, are derived from a disrupted relationship between commensal and harmful microbiota. While the commensal microbiota can foster the development and maturation of the mucosal immune system, its pathogenic counterpart can ruin the immunity, thus accelerating the progression of immunological disease [11]. The gut mucosal immune system, composed of lymphoid tissues, epithelial barriers, and lamina propria (LP) cells, forms a crucial protective barrier that safeguards intestinal integrity [12]. This complex immune network ensures its alignment with host health requirements (Figure 1).

Strands of evidence have highlighted the significant relationship between mucosa‐associated microbiota and immunological diseases [13]. Further research is necessary to reveal the mechanisms through which it can enhance health as well as contribute to disease. Up to now, the majority of studies have focused on assessing the composition of microbiota by 16S rRNA gene sequencing of fecal samples. Given the critical role of mucosa‐associated microbiota, investigating this relationship entails access to intestinal biopsies along with molecular methodologies. Furthermore, the link between microbiota and immunity has not yet been fully clarified. Recently developed methodologies that enable specific mucus‐associated microbiota examination are expected to accelerate progress in this realm. Besides the composition of microbiota, identifying the gene expression also seems to be an endeavor. This is crucial for comprehending the mechanisms by which certain microbiota members colonize this distinctive niche in a manner that triggers diseases. Although the field of mucosal barrier dynamics is in its infancy, it presents promising prospects for the prevention and treatment of diseases.

### 2.2 PRR Signaling Networks

**FIGURE 2:**
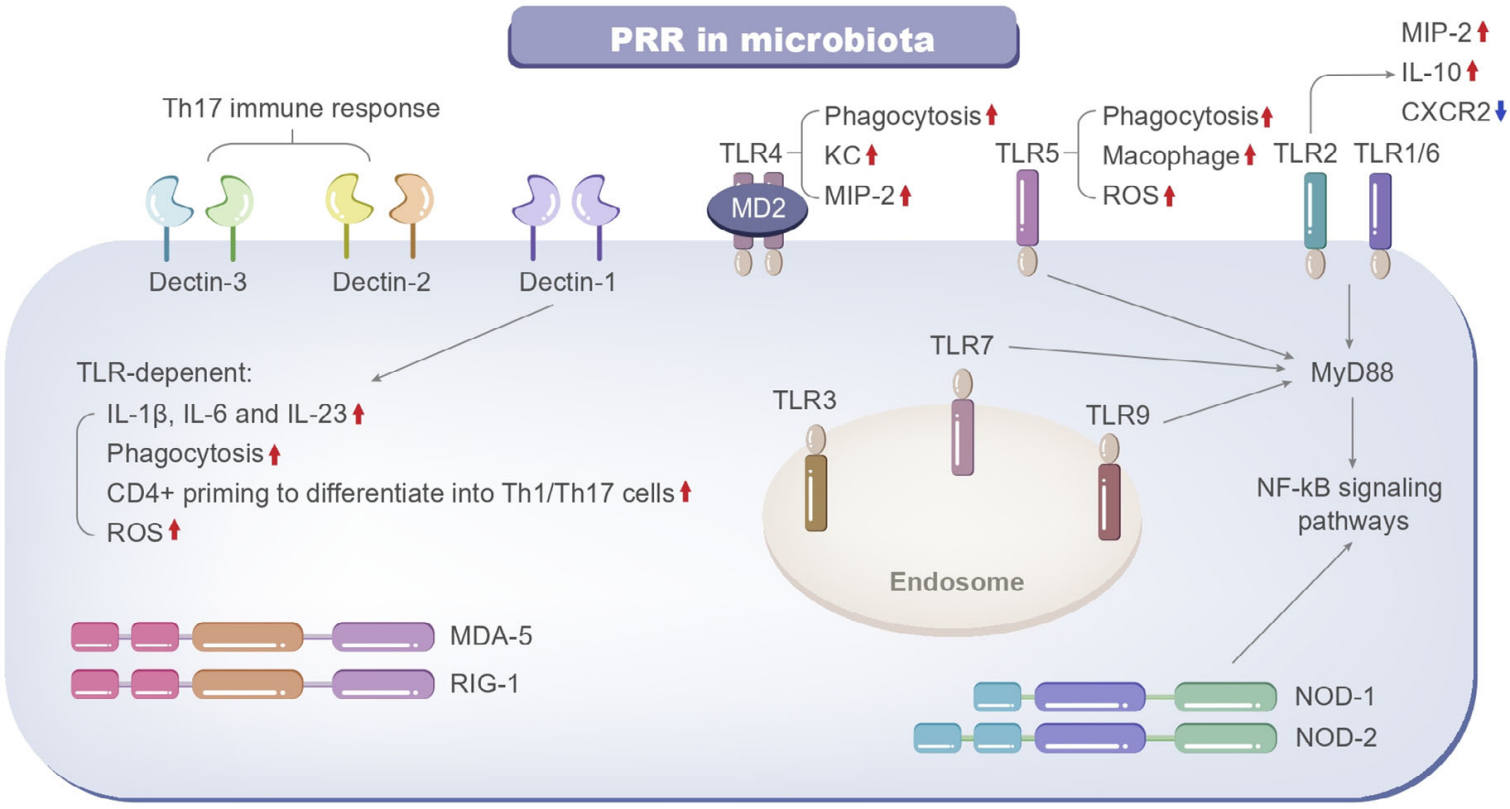
PRR signaling networks. Microbial cell wall components and metabolites interact with the host via PRRs, and thus modulating host immune responses. *Abbreviations*: MD2, myeloid differentiation factor 2; NF‐κB, nuclear factor‐kappa B; NOD, nonobese diabetic; PRR, pattern recognition receptors; ROS, reactive oxygen species; TLR, Toll‐like receptors.

PRRs are a category of receptors that can directly identify the specific molecular structures on the surfaces of damaged or senescent cells, apoptotic host cells, and pathogens [14]. These receptors comprise lipids, nucleic acids, and proteins, including molecules like lipopolysaccharides (LPS), bacterial DNA, and lipoteichoic acid (LTA) (Figure 2).

PRRs serve as a linkage between innate immunity and adaptive immunity. By identifying and binding to specific ligands, PRRs can induce nonspecific defense mechanisms, such as antitumor and antimicrobial activities, as well as other protective immune effects. PRRs are present on a broad range of both immune and nonimmune cells [15]. These receptors can detect various viral and bacterial molecules, including double‐stranded RNA, peptidoglycan, CpG DNA, and LPS. PRRs consist of Toll‐like receptors, C‐type lectin receptors, nucleotide‐binding oligomerization domain‐like receptors, DNA‐sensing molecules, and retinoic acid‐inducible gene I‐like receptors [16].

Identifying commensal microbiota by PRRs is crucial for retaining the interactions and homeostasis between the host and microbiota. The presence of PRRs on multiple cell types renders them a complex and novel target for modulating host–microbe signaling. Microbial cell wall components and metabolites interact with the host via PRRs, and thus modulate host immune responses. PRRs located on the cell membrane and within the cytoplasm are fundamentally composed of three domains: ligand recognition domains, intermediate domains, and effector domains [17]. These receptors initiate downstream signaling pathways by binding to their respective ligands. The activation of these pathways can lead to a variety of outcomes: recruiting and releasing cytokines, chemokines, hormones, and growth factors; inducing chronic inflammation; creating an inflammatory microenvironment; triggering innate immune killing and subsequent adaptive immune responses, maintaining host microecological balance; and eliminating dead or mutated cells. Pathogen‐associated molecular patterns (PAMPs) are highly conserved molecular structures that are specific to particular types of pathogenic microbiota. These structures include lipids, proteins, and nucleic acids such as LPS, LTA, and bacterial DNA. PAMPs are crucial for the survival of pathogens and typically possess unique molecular or subcellular features that are not present in host cells [18]. As a result, innate immune cells can detect PAMPs through PRRs, distinguish between “self” and “non‐self,” and respond to pathogens and their products.

Understanding the way in which our host immunity reacts to microbiota via PRRs is crucial for unraveling the mechanism of diseases.

### 2.3 Metabolite‐Mediated Epigenetic Regulation

Several factors associated with immunological diseases, especially microbiota and epigenetic modifications, play a critical role in managing these conditions [19]. Interestingly, microbial metabolites seem to generate substrates and enzymatic regulators that modulate epigenetic modifications, such as histone modifications, noncoding RNA expression, and DNA methylation [20]. These epigenetic changes, driven by microbial metabolites, contribute to many diseases through influencing intestinal permeability, inflammation, and immune responses. Furthermore, microbial metabolites can bind to G‐protein‐coupled receptors (GPRs), triggering inflammatory immune responses, leading to microbiota dysbiosis [21]. Given that three primary categories of metabolites, namely, SCFAs, tryptophan (Trp), and bile acids (BAs) metabolites, have been recognized within the microbiota, we will elucidate the metabolic pathways of SCFAs, Trp, and BAs in various microbiota.

Macrophages (Macs) exhibit heterogeneity. Their functions and phenotypes are influenced by the surrounding microenvironment. These cells are typically divided into two main types, inflammatory Macs (iMacs) and tolerogenic Macs (tMacs) [22]. iMacs participate in inflammatory immune responses, while tMacs repress inflammation and maintain homeostasis by producing high levels of TGF‐β and IL‐10. In resting intestine, mature resident Ly6Clow/‐CX3CR1hiMHCIIhi tMacs, which are derived from inflammatory Ly6Chigh monocytes/Macs, reside within the LP or the muscle layer to preserve intestinal stability. LP Macs can be further divided into mucosal and submucosal subsets [23]. Metabolites derived from the microbiota can enhance the differentiation of iMacs into tMacs.

**FIGURE 3:**
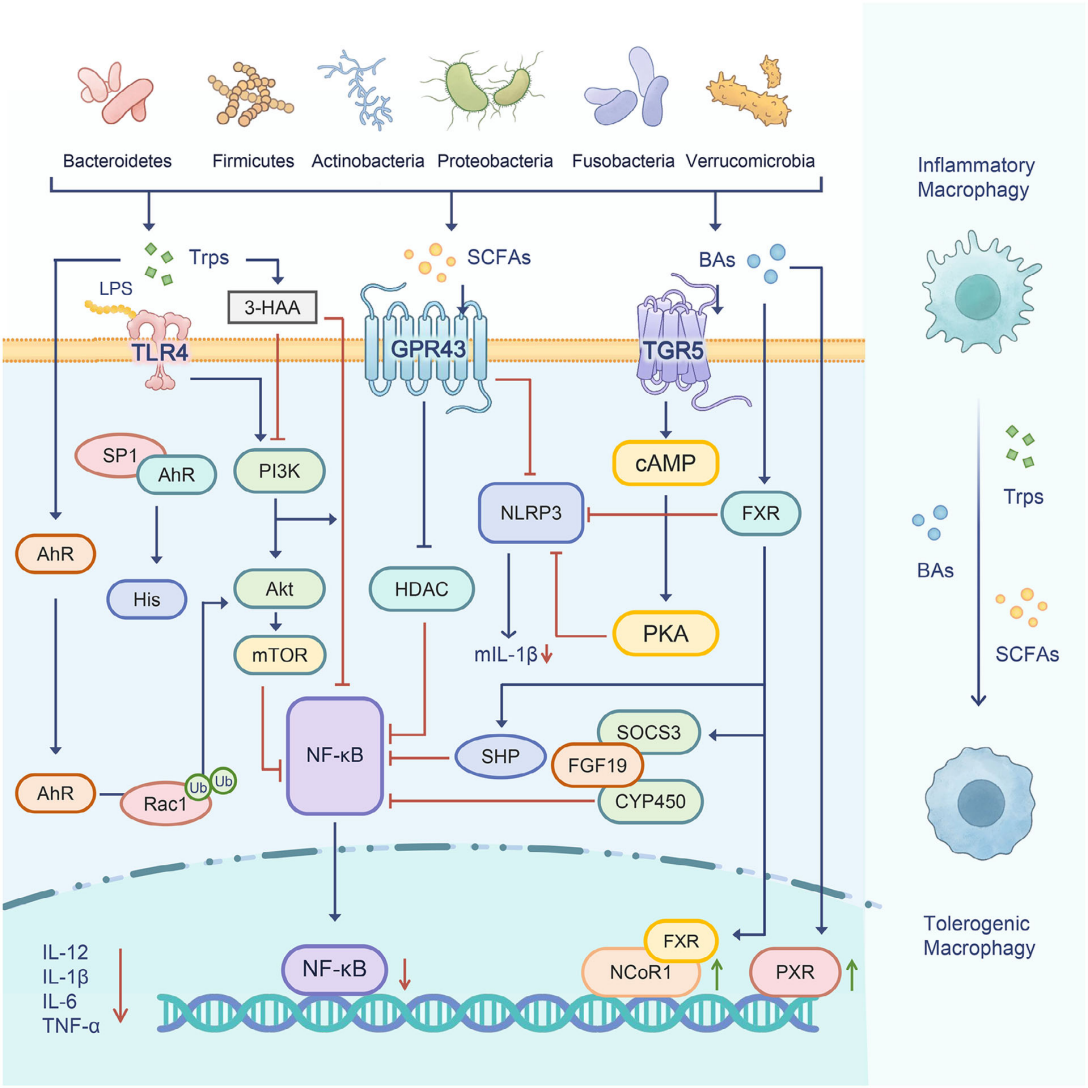
Metabolite‐mediated epigenetic regulation in microbiota. Microbiota promotes the differentiation and function of tolerogenic macrophages through the receptors expressed in the macrophages such as tryptophan metabolites through the AhR nuclear receptor, short‐chain fatty acids through membrane receptors such as GPR43, and bile acid metabolites through the TGR5 membrane receptor and/or FXR nuclear receptor. *Abbreviations*: AhR, aryl hydrocarbon receptor; Akt, protein kinase B; BA, bile acids; CYP450, cytochrome P450; cAMP, adenosine monophosphate; FXR, farnesoid X receptor; FGF, fibroblast growth factor;GPR, G‐protein coupled receptor; HAA, 3‐hydroxyanthranilic acid; SP1, specificity protein 1; His, histamine; HDAC, histone deacetylase; mTOR, mammalian target of rapamycin; NF‐κB, nuclear factor‐kappa B; NLRP, NOD‐like receptor thermal protein domain associated protein; PXR, pregnane X receptor; NCOR1, nuclear receptor corepressor 1; PKA, protein kinase A; PI3K, phosphatidylinositol 3 kinase; Rac1, ras‐related C3 botulinum toxin substrate 1; SCFAs, Short chain acids; SOCS3, suppressor of cytokine signaling 3; TGR, Takeda G protein‐coupled receptor; TLR, Toll‐like receptor; Trp, tryptophan.

Microbiota‐derived metabolites are key in driving the differentiation and function of tMacs through various receptors, SCFAs, as epigenetic substrates, exert anti‐inflammatory effects via regulating the epigenetic modifications. And they also interact with cell surface receptors, with the ileum epithelial cells performing proinflammatory effects. SCFAs interact with membrane receptors like GPR43, Trps engage the AhR nuclear receptor, and BAs connect with the TGR5 membrane receptor and/or FXR nuclear receptors [24] (Figure 3).

Furthermore, exposure to drugs such as analgesics and antibiotics can disturb the balance of the microbiome, particularly during early life stages. Notably, drugs can be metabolized either directly or indirectly by microbes. This interaction can potentially lead to metabolic disruptions. It also demonstrates that microbiota–epigenetics may play a role in metabolic diseases, driven by metabolites from diverse bacterial groups, including Bacteroidetes, Firmicutes, Actinobacteria, Proteobacteria, Fusobacteria, and Verrucomicrobia.

The dynamic interplay between the epigenetic mechanisms and microbiota has a significant impact on health. Microbiota is now increasingly viewed as an environmental factor impacted by epigenetic modifications. However, the intricate connections between the microbiota and immunity are still not well understood and need further exploration.

## 3 Phylum‐Specific Immunomodulatory Properties

The microbiota is pivotal for our health. It includes different phyla like Bacteroidetes, Firmicutes, Actinobacteria, Proteobacteria, Fusobacteria, and Verrucomicrobia. The relationship between microbiota and the immune system is two ‐ way, with immune signals also shaping microbiota makeup and function. For instance, cytokines released during immune responses can change the gut environment, promoting the growth of specific microbial species. This interaction highlights the need for a healthy microbial community to keep the immune system balanced. The biological functions of the microbiota are diverse and essential for maintaining human health. It protects the immune system, helping immune cells mature and establishing tolerance. It also maintains the gut barrier, preventing pathogen movement and increasing antimicrobial peptide production. The microbiota performs essential structural functions by forming the protective mucus layer and regulating tight junctions between intestinal cells. It synthesizes vital vitamins, degrades dietary fibers, and generates anti‐inflammatory SCFAs. The microbiota also modulates the balance of inflammatory cytokines, thereby influencing immune responses [25].

### 3.1 Bacteroidetes

Bacteroidetes are a group of Gram‐negative, obligate anaerobic, and non‐spore‐forming bacteria. They constitute a significant part of the microbiota. These bacteria can metabolize numerous glycans. They are capable of breaking down carbohydrates to produce SCFAs [26]. The Bacteroidetes phylum can be found in various habitats, including soil, the ocean, and freshwater.

#### 3.1.1 Disease Associations

Bacteroidetes showed its abilities in many immunological diseases. Interestingly, *Bacteroides uniformis* is a rising star in the microbiota–immune system.

IBD is closely related to gut dysbiosis and can potentially lead to colitis‐associated malignancies. A structural analysis of a capsular polysaccharide called BUCPS1B from *B. uniformis* ATCC8492 revealed its beneficial effects on the DSS‐induced colitis mouse model. Moreover, BUCPS1B reshaped the gut microbiota by increasing the probiotic *A. muciniphila* level and altered the gut metabolic profile to enhance phenylalanine and SCFA metabolism [27]. A recent study investigated the effects of *B. uniformis* JCM5828 and its metabolites on C57BL/6J mice with colitis induced by DSS. They revealed that treatment with *B. uniformis* significantly mitigated colitis deterioration and reestablished the expression of proteins critical for mechanical and immune barrier functions. Moreover, *B. uniformis* increased the beneficial bacteria abundance [28]. While some studies have highlighted the potential of *Bacteroides fragilis* to trigger inflammation, a recent investigation has revealed its protective function in T1D. Specifically, *B. fragilis* was demonstrated to induce the activation of iNKT cells and M2 Macs in nonobese diabetic (NOD) mice, thereby reducing the incidence of T1D diabetes [29]. In another study, the Bacteroidetes phylum also exhibited its protective effect in diabetes. Treatment with *Prevotella histicola* in NOD mice postponed the development of T1D diabetes [30]. Furthermore, some research also revealed that *Prevotella copri* exacerbated RA [31]. A study found *P. copri* could combine with a high‐fiber diet (HFD) and thus exacerbate RA [32]. In an experiment involving two arthritis models, *P. copri* showed proinflammatory ability toward RA [33]. Another trial has also suggested that *P. copri* potentially plays a role in the progression of RA, both in its preclinical phase and in the subsequent development of synovitis [34].

#### 3.1.2 Molecular Effectors

The Bacteroidetes phylum is essential for human health. SCFAs from bacterial fermentation of dietary fibers are crucial for gut homeostasis.

SCFAs connect host nutrition with immunity. They are key energy sources for intestinal epithelial cells (IECs) and influence IEC functions in various ways. SCFAs widely affect human health, covering immunity, energy metabolism, and boosting intestinal barrier function. Moreover, they can impact the central nervous system (CNS) through the gut–brain axis, enhancing cognition and memory [35]. SCFAs from Bacteroidetes impact IEC functions through different mechanisms. This includes influencing their growth, development, and the activities of specific subpopulations like enteroendocrine cells.

Bacteroidetes can affect the inflammatory response by regulating the immune signaling pathway. For example, *B. uniformis* significantly regulated key proteins in the NF‐κB and MAPK pathways within colonic tissues [28]. Mice that received supplements of Parabacteroides Goldstein or its BA metabolite, 7‐keto‐lithocholic acid (7‐keto‐LCA), exhibited diminished aspirin‐induced damage to the gut barrier and intestinal niche. Notably, 7‐keto‐LCA enhanced intestinal epithelium repair by inhibiting the activity of the intestinal BA receptor FXR. It has been confirmed that 7‐keto‐LCA acts as an FXR antagonist, facilitating Wnt signaling and thereby supporting the self‐renewal of intestinal stem cells [36]. Polysaccharide A (PSA) is a capsular carbohydrate from the *B. fragilis*. It has shown conspicuous efficacy in alleviating various rodent models of inflammatory diseases via the activation of downstream suppressive CD4^+^ T cells. Moreover, PSA stimulated immune‐regulatory protein expression and enhanced the expression of genes such as IL‐2, STAT1, and STAT4. These actions collectively play a crucial role in modulating cytokine production and immune responses [37].

In summary, the role of Bacteroidetes in strengthening the intestinal barrier and regulating inflammation offers promising therapeutic avenues. Future research should focus on harnessing the molecular mechanism of Bacteroidetes’ potential for treating immunological diseases.

#### 3.1.3 Functional Outcomes

As microbiota commensals, Bacteroides fulfils various functions. They act as a defense against pathogens and provide essential nutrients to other microbiota.

Previous studies demonstrated that mucin‐type O‐glycans are crucial factors in their beneficial interactions and directly influence the interaction between Bacteroides and host tissues [38]. *Bacteroides thetaiotaomicron* VPI‐5482 strain possesses polysaccharide utilization loci (PULs), enabling it to dispose of various glycans, including those derived from diet and host sources. *B. thetaiotaomicron* used PULs to find O‐glycans efficiently when plant‐derived polysaccharides are scarce [39]. Mucins are key to the fitness and stability of Bacteroides. The interaction between *B. thetaiotaomicron*, mucins, and breakdown affects the genetic elements for outer capsule synthesis. *B. thetaiotaomicron* strains, lacking adhesive organelles, used outer membrane glycan‐binding proteins to attach to food particles, mucus layers, and exfoliated epithelial cells. *B. thetaiotaomicron* can easily switch to using host‐derived polysaccharides when dietary ones are scarce, showing a flexible capacity for glycan foraging. Like *B. thetaiotaomicron*, *B. fragilis* had the genetic setup to break down and use glycans, including mucin‐type O‐glycans, to make capsular polysaccharides, which is crucial for their colonization and maintenance in the gut [40]. This helps maintain the gut microbiome's stability, especially during nutritional shortages [41]. Mucins also promote microbial colonization by providing glycans as substrates for bacterial residents of the gut, including *Bacteroides*. This, in turn, affects the distribution of these bacteria and provides them with an additional nutritional source. Consequently, mucin glycans in Bacteroides are thought to play a crucial role in shaping and sustaining bacterial communities across the gut microbiome [13]. *B. fragilis* is a crucial therapeutic method in TB infection. Research found that *B. fragilis* was a direct regulator of lncRNA‐CGB, and oral administration of *B. fragilis* enhanced the expression of lncRNA‐CGB and promoted anti‐TB immunity [42]. In models of nonalcoholic steatohepatitis (NASH), *Parabacteroides distasonis* showed potential in repairing gut barrier function, thereby decreasing serum LPS levels and the expression of proinflammatory cytokines in the liver [43]. However, enterotoxigenic *B. fragilis* (ETBF) demonstrated its pathogenic risk. The presence of ETBF has been widely documented in CRC [44 ^,^ 45]. Specifically, ETBF could serve as a diagnostic biomarker for CRC [46]. A study eradicated its involvement in damaging the intestinal barrier and accelerating the progression of CRC [47]. ETBF was considered a potential driver of CRC. It stood out as the sole pathobiont detected at the early adenoma stage [48]. Also, the presence of ETBF is linked to improved outcomes in patients with CRC following curative resection. As such, ETBF may hold clinical significance as a potential prognostic indicator in CRC [49, 50]. Microbiota constitutes one of the intestinal barriers and plays a crucial role in forming biofilms, contributing to intestinal function, and shaping the environment for intestinal cells. Another study demonstrated that *B. fragilis* is a microbial species that strengthens the intestinal epithelial barrier [51].

Bacteroides species exhibit remarkable adaptability in glycan utilization and host interaction, highlighting their importance in health and disease. Understanding these mechanisms offers insights into potential therapeutic strategies for treating immunological diseases.

### 3.2 Firmicute

Phylum Firmicutes includes a wide range of species [52], from pathogens like *Staphylococcus aureus*, *Streptococcus pneumoniae*, *Listeria monocytogenes*, and various *Clostridium* species, to beneficial and harmless species such as *Lactobacillus lactis*, *Staphylococcus carnosus*, *Streptococcus thermophile*, and *Faecalibacterium prausnitzii*.

#### 3.2.1 Disease Associations

Firmicutes offers a new strategy for combating diseases. Bacillus repressed the Th2 response and stimulated Macs to enhance Th1 immune responses [53]. It attenuated colitis symptoms, suppressed immune response, and improved the imbalanced gut microbiota in colitis mice.

Notably, *Clostridium butyricum* potentially becomes a rising star in treating colitis. The protective effects of *C. butyricum* extended beyond the bacterium itself; its culture and supernatant also demonstrated significant therapeutic potential. Research showed that both the culture and supernatant of *C. butyricum* exert inhibitory effects on inflammatory CRC in mice [54]. In the active immunization model of experimental autoimmune encephalomyelitis (EAE), epsilon toxin (ETX)‐induced EAE resulted in demyelination within the thalamus, corpus callosum, brainstem, cerebellum, and spinal cord, resembling that observed in MS. Additionally, transcriptional profiling of CNS endothelial cells identified ETX‐induced genes implicated in combating CNS immune privilege [55].

Collectively, these findings support that Firmicutes are potentially indispensable in immunological diseases, capable of triggering proinflammatory and anti‐inflammatory factors.

#### 3.2.2 Molecular Effectors

The relationship between Firmicutes and immunological diseases has garnered significant attention in recent years. The molecular mechanisms underlying Firmicutes have shown promise in modulating immune responses through various pathways.

SCFAs produced by the phylum Firmicutes play a critical role in regulating Firmicutes and immunological diseases. Specifically, butyric acid can repress histone deacetylase (HDAC) and increase FoxP3 expression, thereby enhancing the activity of Tregs. SCFAs can also reduce the NF‐kB inflammatory pathway through PPAR‐γ. *C. butyricum* was found to suppress the activity of HDAC and inhibit TLR4‐mediated phosphorylation of NF‐κB [56]. *C. butyricum*, too, strongly promoted the expression of key tight junction proteins like claudin‐3, ZO‐1, and occludin. Mono‐colonizing germ‐free mice with *Clostridium sporogenes* altered the phenotypes of colonic mucosal immune cells, including increased IL‐22 gene expression, as well as increased transcriptionally active colonic tuft cells abundance and Foxp3+ Tregs [57]. *Bacillus licheniformis* MCC2514 was also identified as an immune modulator capable of regulating Foxp3 expression in conditions of elevated disease. This strain demonstrated significant potential for modulating immune responses, which is particularly relevant in managing inflammatory conditions [58]. However, it has been suggested that *Enterococcus faecalis* in the presence of butyrate could enhanced inflammasome activation in Macs by inhibiting HDAC. This mechanism may lead to dental inflammatory diseases like apical periodontitis [59]. In another experiment, it was found that *Enterococcus faecium* B6 produced a significant bioactive metabolite called tyramine, which potentially activated PPAR‐γ, contributing to lipid accumulation, inflammation, and fibrosis in the liver [60]. Firmicutes could also induce the release of molecules from the IECs surface, thereby enhancing the integrity of the gut barrier. The intervention with *Bacillus siamensis* markedly activated the signaling pathways of TLR2 and TLR5. This activation ultimately repressed the p38 and NF‐κB pathways, thus significantly enhancing intestinal barrier function [61]. A research study demonstrated that *F. prausnitzii* exerted therapeutic effects on RA by modulating IL‐17‐producing cells. Additionally, it altered the composition of the gut microbiota and SCFA levels in the RA mouse model [62]. Another study revealed that *Bacillus coagulans* significantly altered TNF‐α, IL‐1β, and IL‐22 levels. It also helped keep tight junctions and mucin proteins at proper levels, helping gut barrier function recovery and thus augmenting immunity [63].

The molecular mechanisms of Firmicutes hold great potential for developing new therapies for immunological diseases. However, more studies are needed to fully grasp the complex interactions within these mechanisms and to create more targeted treatment approaches.

#### 3.2.3 Functional Outcomes

The Firmicutes, particularly *C. butyricum*, are well known for their various roles in preserving intestinal health, showing potential in adjusting immune responses, improving metabolic activities, and enhancing immunity. Understanding this phylum's biological functions is key to treating immunological diseases.

Firmicute achieves its biological functions by keeping the balance in the body and lessening inflammation. A study revealed that supplementation of *C. butyricum* alleviated vascular inflammation in diabetic mice [64]. Another study provided valuable insights into the mechanisms by which *C. butyricum* modulates tumorigenesis and enhances the efficacy of chemotherapy and immunotherapy. Specifically, treatment of CRC with *C. butyricum* led to MYC degradation through enhanced proteasome‐mediated ubiquitination, and thus effectively mitigated MYC‐driven 5‐FU resistance and boosted anti‐PD‐1 immunotherapy effect [65]. Administration of *C. butyricum* orally following gastrectomy conferred several therapeutic benefits. Specifically, it reduced early postoperative inflammation, augmented immune function, restored microbiota homeostasis, increased SCFAs, decreased complications incidence, and ultimately enhanced early patient recovery [66]. These findings unveiled the multifaceted therapeutic potential of *C. butyricum* in addressing a range of immunological diseases. However, some research has also demonstrated that *Clostridium* can lead to adverse outcomes, including *Clostridium innocuum* infection, and even death clinical trial revealed that this infection can potentially result in poorer clinical remission in UC [67]. Analogously, other experiments also unveiled adverse effects of *Clostridium* [68, 69, 70].

Firmicute has a good nutritional status; it can regulate metabolism, modulate the immune system, and maintain intestinal barrier function. *C. butyricum* plays a significant role in modulating purine metabolism and enhancing water–salt balance, which helps alleviate inflammatory responses. It also created a more favorable intestinal microbiota environment that encourages the proliferation of beneficial bacteria. These combined effects lead to increased metabolic activity, ultimately promoting better health and growth of the organism [71]. *Clostridium tyrobutyricum* has shown promise in improving lipid metabolism disorders caused by an HFD, preserving the integrity of the intestinal barrier, and altering the structure of the microbiota [72].

Firmicute demonstrates significant potential in maintaining intestinal homeostasis and overall well‐being. Its capacity to engage with the immune system presents novel therapeutic avenues for multiple diseases. However, additional research is necessary to fully exploit its advantages while reducing possible risks.

### 3.3 Actinobacteria

Actinobacteria is a phylum of Gram‐positive bacteria known for their high genomic and metabolic diversity. It plays a key role in immune regulation through complex molecular and biological functions [73]. These bacteria interact with the host immune system through various mediators, such as metabolites, cell wall components, and secreted proteins, thus influencing both innate and adaptive immune responses.

#### 3.3.1 Disease Associations

Probiotics and Trp metabolites are becoming increasingly important in treating immunological diseases. Indole‐3‐carboxaldehyde is a metabolite of Trp derived from *Bifidobacterium*. Research revealed that it could activate the AHR immune signaling pathway, which has been shown to alleviate atopic dermatitis symptoms [74]. *Bifidobacterium animalis* subsp *lactis* CCFM1274 produced high levels of indole‐3‐carboxaldehyde. In addition, in the ovalbumin (OVA)‐induced allergic asthma mice model, the animals were subsequently treated with *B. animalis* subsp *lactis* CCFM1274. The results indicated that this treatment attenuated body weight loss and reduced mortality in mice [75]. A research demonstrated that *Bifidobacterium pseudolongum* exerted a protective effect against DSS‐induced colitis through the production of R‐equol. Notably, this process is significantly enhanced in the presence of indole‐3‐acetic acid [76]. In a randomized controlled trial, infants exposed to HIV who received *Bifidobacterium infantis* supplementation exhibited significantly enhanced immune function compared with those administered a placebo [77].

#### 3.3.2 Molecular Effectors

Actinobacteria, a major phylum in the microbiota, are key players in shaping immune responses. They interact with the host immune system via various mechanisms, such as producing metabolites, expressing surface molecules, and engaging in direct cell‐to‐cell contacts.

SCFAs bind to GPRs [78, 79], inhibiting HDACs to enhance Treg differentiation and suppress proinflammatory Th17 cells [80, 81]. Researchers analyzed the microbiota in individuals with T1D and T2D. They also assessed the correlation between the GPR41 and GPR43 gene expression levels. They found the abundance of *Bifidobacterium longum* and *Faecalibacterium prausnitzii* markedly reduced in patients [78]. This study uncovered an association between the microbiota and GPR, highlighting a potential link between microbial composition and host immune‐related pathways mediated by these receptors. A randomized controlled trial demonstrated that sulforaphane enhanced insulin sensitivity by modulating the SCFAs–GPR–GLP1 signaling axis [82]. This trial highlighted the pivotal role of SCFAs in engaging GPRs to stimulate GLP‐1 secretion, thereby improving metabolic homeostasis. In addition, this trial also underscored the interplay between microbiota and host metabolic pathways, positioning the SCFAs–GPR–GLP1 axis as a key therapeutic target for addressing insulin resistance. A study demonstrated that *B. pseudolongum* effectively attenuated nonalcoholic fatty liver disease (NAFLD), development by secreting acetate. This binds to hepatic GPR43 and thus inhibits the oncogenic IL‐6/JAK1/STAT3 signaling pathway [83].

In summary, current studies reveal the notable influence of Actinobacteria on host immunity and metabolism. The regulation of SCFAs–GPR signaling axes offers potential therapeutic strategies for immune and metabolic disorders. However, more research is needed to fully understand the complex interactions among specific microbial strains, their metabolites, and host pathways. This knowledge will facilitate the development of targeted microbial interventions and personalized therapeutic approaches.

#### 3.3.3 Functional Outcomes

Actinobacteria phylum is essential for our biological functions. They maintain a balance between tolerance and defense, impacting various immune system pathways. Understanding their functions can help manage immune disorders and improve health.

Actinobacteria play a key role in shaping immune responses, striking a balance between tolerance and defense. For example, *B. infantis* boosted FoxP3 expression by inhibiting the PI3K–Akt–mTOR pathway through PD‐L1 [84]. SCFAs produced by Actinobacteria reshaped immunity. SCFAs inhibited the mTOR pathway in dendritic cells (DCs), shifting their metabolism to oxidative phosphorylation. This switch reduced the DC‐driven polarization of Th1/Th17 cells, favoring immunity enhancement [85]. *Bifidobacterium* depletion correlated with adipose tissue inflammation and insulin resistance. Restoring *Bifidobacterium* populations increased IL‐10‐producing Macs [86], ameliorating metabolic dysfunction.

Actinobacteria are capable of regulating immune pathways and reducing inflammation. More research is necessary to utilize their potential in enhancing immunity.

### 3.4 Proteobacteria

Proteobacteria are a group of Gram‐negative bacteria. They are gaining attention for their roles in various immunological diseases [87]. Dysbiosis is often marked by Proteobacteria enhancement, which is linked to immunity. Gaining insights into the Proteobacteria is potentially a key to creating more targeted treatment approaches.

#### 3.4.1 Disease Associations

In a study utilizing an oral *Salmonella*‐based vaccine, researchers observed the reversal of diabetes in NOD mice with both acute and progressive forms of the disease. The *Salmonella*‐based vaccine played a crucial role in modulating the immune response, ultimately leading to the amelioration of diabetes symptoms in the treated mice [88]. A study demonstrated that the gut commensal *Escherichia coli* exacerbated HFD‐induced obesity and insulin resistance in mice, thereby contributing to T2D development [89]. This study highlighted the role of gut microbiota in modulating metabolic disorders, specifically linking the presence of commensal *E. coli* to the progression of diabetes‐related symptoms. The interplay between microbiota and the host immune system thus represents a key mechanism driving the pathogenesis of IBD.

#### 3.4.2 Molecular Effectors

Proteobacteria, including *E. coli* and *Helicobacter pylori*, play a significant role in modulating immunity through various molecular pathways. Proteobacteria could influence immune reactions in ways that impact immunological diseases. *E. coli* CNF1 was deemed vital in CRC development. This is achieved through mechanisms involving oxidative stress‐induced DNA damage and increased intestinal permeability [90]. The molecular mechanisms involved in increased proinflammatory cytokines like TNF‐α, IL‐1β, and IL‐6. Proteobacteria LPS activated TLR4. This process aggravated immunological diseases. A study found that *Salmonella Typhimurium* could block IFNβ signaling in Macs. Importantly, this inhibition was found to occur in a manner that was dependent on TLR4 [91]. This research highlighted a specific mechanism through which *S. Typhimurium* could modulate the host immune response to its advantage. Proteobacteria‐derived metabolites, such as TMAO and uremic toxins, activate the NLRP3 inflammasome. TMAO promoted caspase‐1 cleavage and IL‐1β secretion in Macs, linking dysbiosis to immunological diseases. Studies have shown that H. pylori infection could drive M1 Mac polarization and gastric inflammation development via the inflammasome [92]. However, this Proteobacteria also employed ROS/RNS to inhibit NLRP3, steering Mac polarization toward chronic inflammation and gastric issues [93].

The molecular mechanisms and evolutionary paths of Proteobacteria are complex. Studying these processes helps us understand the functional and evolutionary aspects of Proteobacteria.

#### 3.4.3 Functional Outcomes

Proteobacteria have sparked substantial interest in recent years due to their complex biological functions in modulating immunity.

Commensal Proteobacteria can reduce intestinal epithelial inflammation and enhance tight junction barrier function [94]. When microbiota becomes imbalanced, certain Proteobacteria like *Enterobacteriaceae* can overgrow and break down protective mucin layers. This breakdown compromises the intestinal lining's integrity, potentially allowing toxins to enter the bloodstream—a condition known as systemic endotoxemia. Researchers demonstrated that *Helicobacter hepaticus* CdtB toxin specifically damaged the mucous barrier in mouse colons. These insights into how Proteobacteria interact with host immune defenses have opened new avenues for developing targeted therapies aimed at restoring microbial balance and gut health [95]. In another study, Enterohemorrhagic *E. coli* was seen to destroy intestinal barrier integrity in canine stem cell‐derived monolayers [96]. This finding highlights the pathogenic impact of this bacterium on intestinal barrier function in a preclinical setting.

Proteobacteria antigens shape adaptive immunity. Proteobacteria can also serve as an adjuvant to augment the adaptive immune responses by supplementing the components of vaccines. In a murine model, the *E. coli* LTB26 mutant was found to reinforce immune responses against the rotavirus antigen VP8 [97]. Specifically, it enhanced both Th1 and Th2 immune responses, demonstrating its potential to bolster adaptive immunity in this context.

*Helicobacter* species are detected in wild mice and have demonstrated conferring protection against pathogenic challenges in settings where adaptive immune responses are absent or compromised [98]. This research highlighted the capacity of commensal *Helicobacter* species to modulate innate immune pathways and limit microbial colonization, even when adaptive immunity is nonfunctional. It also underscored the nuanced role of these bacteria in shaping host–microbe interactions outside the context of conventional immunity. *E. coli* employed a leucine zipper‐based methodology to produce soluble T‐cell receptors, thereby enhancing adaptive immunity [99]. Proteobacteria can affect insulin resistance in *E. coli*, and thus exacerbate HFD‐induced insulin resistance in mice [89].

The biological functions of Proteobacteria have a complex relationship with the immune system. This offers promising prospects for immune modulation. Their potential as immune adjuvants is worth further exploration. This could lead to new therapeutic strategies in human medicine.

### 3.5 Fusobacteria

Fusobacteria are Gram‐negative, strictly anaerobic microbes that mainly live in the mouth, gut, and female reproductive system. They are usually present in small numbers and coexist peacefully with us. However, some species like *Fusobacterium nucleatum* and *Fusobacterium periodonticum* can cause trouble. They are linked to diseases related to microbial imbalance, such as periodontal disease [100], CRC [101], IBD, and autoimmune disorders. These bacteria can cause problems by triggering chronic inflammation, damaging the protective barrier of our body surfaces, and interfering with the normal function of immune cells.

#### 3.5.1 Disease Associations

Fusobacteria are closely associated with numerous immunological diseases. Notably, *F. nucleatum* aggravated RA through FadA‐containing outer membrane vesicles (OMVs). In a mouse model of collagen‐induced arthritis, *F. nucleatum* alleviated RA by translocating OMVs containing the virulence factor FadA into joint tissues, thereby triggering local inflammatory responses [102]. A study demonstrated that oral administration of F. nucleatum led to a worsening of UC in mice, an effect attributed to the secretion of the virulence adhesin FadA [103]. Extracellular vesicles derived from *F. nucleatum* exacerbated experimental colitis through modulating autophagy pathways [104]. Another study also indicated that examining and eradicating *F. nucleatum* may represent a promising approach for the surveillance and prevention of IBD [105].

#### 3.5.2 Molecular Effectors

Fusobacteria, particularly *F. nucleatum*, drive immunological dysregulation through multifaceted molecular pathways that intersect host–microbe interactions and immune signaling.

*F. nucleatum* expressed surface adhesins like Fap2, RadD, and LPS that activated TLR2/4 on DCs and Macs, driving proinflammatory cytokine production. This chronic inflammation disrupted mucosal tolerance and promoted autoantibody generation in susceptible hosts. *F. nucleatum* drove inflammatory and antiapoptotic signaling in CRC cells through the release of ADP‐heptose and subsequent activation of the ALPK1/TIFA pathway [106]. *F. nucleatum*‐derived OMVs elicited periodontitis by inducing a hyperactive host immune response [107]. *F. nucleatum* has become a key player in tumorigenesis and metastasis through diverse mechanisms [108]. These mechanisms encompassed the modulation of immune responses, exemplified by the action of Fap2 and RadD proteins that suppress antitumor immunity, as well as the promotion of cell proliferation, induction of DNA damage, and facilitation of the epithelial–mesenchymal transition. Furthermore, compelling research suggested that *F. nucleatum* may aggravate cancer progression. Research revealed that the adhesin RadD facilitated the tumor colonization of *F. nucleatum* and accelerated colorectal carcinogenesis [109]. Intratumoral *F. nucleatum* was also found to recruit tumor‐associated neutrophils to promote gastric cancer progression and immune evasion [110]. Additionally, *F. nucleatum* interacted with intestinal metabolites to reshape the tumor microenvironment, further driving oncogenesis. *Fusobacteria* produce SCFAs, enhancing their immunosuppressive function [111].

These mechanisms collectively promote a proinflammatory milieu, disrupt immune homeostasis, and facilitate chronic disease progression. Targeting *Fusobacteria*–host receptor interactions or metabolite pathways represents a promising therapeutic frontier.

#### 3.5.3 Functional Outcomes

Fusobacteria, especially *F. nucleatum*, contribute to immune system dysregulation through complex molecular pathways that involve both host–microbe interactions and immune signaling processes.

These microbiota elements play a key role in modulating immune responses, creating a microenvironment conducive to tumor development. Notably, *F. nucleatum* has been shown to bind directly to the TIGIT immune checkpoint receptor, effectively enabling cancer cells to bypass immune surveillance through this mechanism [112]. This interaction essentially acted as a shield, protecting cancer cells from immune system attacks and allowing tumors to grow unchecked. Furthermore, *F. nucleatum* weakened the body's natural cancer defenses by triggering CEACAM1 activity. This double whammy significantly hampered the immune system's ability to fight cancerous growths effectively [113]. Research indicated that Fusobacteria contributed substantially to inflammatory processes within the body. These bacteria appeared to activate cellular pathways that stimulate the production of inflammation‐promoting signaling molecules, particularly IL‐6 and IL‐8. This biological activity exacerbated inflammatory conditions associated with multiple health disorders. Scientists have discovered that this specific bacterium modifies IL‐17 protein levels through interactions with metabolite receptors, effectively transforming the intestinal ecosystem into one that favors chronic inflammation. This inflammatory shift is thought to create conditions conducive to tumor development and disease advancement in CRC patients [114, 115, 116, 117]. This inflammatory milieu not only facilitates tumor development but also propels the entire disease trajectory. Moreover, Fusobacteria can modulate immune system activities, especially impacting Tregs that are critical for governing immune responses [118]. Fusobacteria helped tumors evade immune detection by suppressing the local immune environment. They reduced cancer‐fighting T cell activity while enabling tumor growth. Current research prioritizes mapping their precise mechanisms and identifying vulnerable points for therapies that block these effects without harming beneficial bacteria.

### 3.6 Verrucomicrobia and Immunological Diseases

The phylum Verrucomicrobia encompasses a broad range of Gram‐negative bacteria that display a variety of cell morphologies, including spherical, rod‐like, and spiral configurations [119]. This cluster encompasses multiple orders, such as Opitutales, Puniceicoccales, and Verrucomicrobiales*. A. muciniphila*, which is part of the Verrucomicrobiales superphylum, is particularly noteworthy. This is mainly due to its distinctive biological attributes, which have made it a focal point in microbiological studies [120]. *A. muciniphila* impacted host health via diverse molecular interactions, such as mucin metabolism, immune regulation, BA conversion, and inhibition of harmful bacteria. The diverse impacts of *A. muciniphila* in different scenarios highlighted the significance of precision medicine, which leverages individual microbiome profiles to attain optimal treatment outcome*s* [121].

#### 3.6.1 Disease Associations

*A. muciniphila* is critical in asthma pathogenesis, progression, and disease severity and is relevant to mucosal immunity and host metabolism. Emerging evidence suggests that *A. muciniphila* holds promise as a therapeutic agent for NAFLD, particularly its inflammatory manifestation known as NASH. Specifically, *A. muciniphila* exerted its protective effects by regulating the polarization of TLR2‐activated γδT17 cells and Macs, thereby attenuating the inflammatory processes characteristic of NASH [122]. *A. muciniphila* exhibited potential efficacy in mitigating OVA‐induced food allergies in mice [123]. Another study further demonstrated that heat‐inactivated *A. muciniphila* effectively alleviated allergic airway inflammation in mice [124]. *A. muciniphila* has also demonstrated its capability in arthritis in preclinical experiments and clinical trials. An experiment revealed that *A. muciniphila* exhibited therapeutic potential in a murine model of acute gouty arthritis. Specifically, this bacterium effectively mitigates the severity of paw edema and concurrently elevates the pain threshold in affected mice [125].

Accumulative evidence has shown that the abundance of *A. muciniphila* is decreased in patients with IBD and animal models with colitis, while administration of *A. muciniphila* showed potential anti‐inflammatory effects by activating related signaling pathways and immune cells. A study revealed a cooperative effect between *A. muciniphila* and *Parabacteroides distasonis*. This strain protected both acute and chronic colitis models by strengthening the epithelial barrier and stimulating group 3 innate lymphoid cells in the colonic mucosa [126]. In cAMP‐responsive element‐binding protein H (CREBH) knockout mice model, *A. muciniphila* and its membrane‐associated proteins have been shown to attenuate intestinal inflammatory stress and facilitate epithelial wound healing through the coordinated actions of CREBH and the miR‐143/145 axis [127].

#### 3.6.2 Molecular Effectors

*A. muciniphila*, a Gram‐negative intestinal bacterium, resides within the gut mucus layer and metabolizes mucin. It has gained significant attention for its potential role in managing immunological diseases. The health benefits associated with this bacterium are attributed to a combination of mechanisms, including interaction with host receptors, modulation of the gut microbiota, and production of bioactive metabolites.

*A. muciniphila* is crucial for gut health as it breaks down mucins, the glycoproteins forming the intestinal mucus layer. This bacterium secretes enzymes such as sialidases and fucosidases, which cleave mucin O‐glycans and release metabolites like acetate and propionate [128, 129, 130]. These SCFAs boost goblet cell activity. This leads to mucus layer replenishment. They also strengthen the epithelial barrier by increasing tight junction proteins. This process ensured proper mucus turnover. It stops pathogens from getting in. At the same time, it creates a good environment for beneficial microbes to live.

*A. muciniphila* interacts with host immune receptors through its membrane‐derived phospholipid, a15:0‐i15:0 phosphatidylethanolamine. This lipid acts as a ligand for the TLR2–TLR1 heterodimer, triggering an anti‐inflammatory response and producing anti‐inflammatory cytokines [131, 132]. This mechanism promotes immune tolerance when bacterial levels are low. However, higher concentrations may lead to inflammation. This shows that its role in diseases like obesity and autoimmune disorders depends on the context. Heat‐inactivated *A. muciniphila* boosts intestinal secretion of insulin‐like growth factor 2. This improves skeletal muscle glucose uptake and reduces muscle atrophy [133]. The bacterium also activates AMPK in adipose tissue. This promotes fatty acid oxidation and increases energy use [134]. Together, these processes improve glucose balance and ease metabolic syndrome. *A. muciniphila* fights off pathogens in two main ways. First, it uses SCFAs to acidify the gut environment. Second, it releases nonacidic bioactive compounds. These compounds mess with pathogen virulence factors, like biofilm formation and epithelial adhesion in *S. typhimurium* [135]. This action lowers the chance of pathogens setting up shop and causing systemic infections. *A. muciniphila* has effects on many levels. Locally, it breaks down mucins. This action influences metabolism and immunity throughout the body. This bacterium engages with host receptors, alters microbial communities, and secretes bioactive compounds.

These capabilities position it as a crucial species for maintaining health. Future research should prioritize understanding strain‐specific variations and developing more effective delivery methods.

#### 3.6.3 Biological Functions

*A. muciniphila* plays a multifaceted role in immune regulation and host–microbe homeostasis. It helps strengthen the gut barrier, balance the immune system, support metabolic functions, and resist pathogens.

*A. muciniphila* triggers mucus layer renewal by degrading mucin glycoproteins, thus reinforcing gut barrier integrity [136]. *A. muciniphila* helps generate SCFAs. SCFAs interact with immune cell GPRs, boosting Treg differentiation and curbing proinflammatory cytokines like TNF‐α and IL‐6 to strengthen anti‐inflammatory responses. *A. muciniphila* also directly interacts with the immune system via cell envelope components such as Amuc_1100 and extracellular vesicles [137]. These structures activate TLR2 and TLR4 on DCs and Macs, initiating a balanced immune response that defends against pathogens while tolerating commensals [131, 138, 139].This interaction enhances the secretion of immunomodulatory cytokines like IL‐10 and TGF‐β, fostering a tolerogenic environment. Notably, *A. muciniphila* correlates with increased mucosal IgA production [140], which reinforces gut barrier defense by neutralizing pathogens and shaping microbial community structure. In cancer immunotherapy, *A. muciniphila* has been linked to enhanced efficacy of PD‐1 checkpoint inhibitors, likely through its ability to stimulate CD8^+^ T cell infiltration into tumors [141, 142].

Overall, *A. muciniphila* exemplifies a keystone symbiont whose immunomodulatory functions span mucosal reinforcement, metabolic regulation, and systemic immune tuning, positioning it as a promising therapeutic target for immune‐related disorders. Future research should prioritize strain‐specific interventions, EV‐based therapies, and clinical trials to validate their efficacy in human immune disorders.

## 4 Translational Development Pipeline

In microbiota–immunity interactions, the translational development pipeline is a critical link that connects fundamental studies to clinical applications. This procession commences with target identification. Organoid screening subsequently takes place, employing organoid models to replicate the intricate environments of human tissues and organs. This enables high‐throughput screening of potential therapeutic agents in a context that closely mimics human physiology. Animal validation then plays a key role in evaluating efficacy and safety in vivo, generating essential preclinical data. Ultimately, the design of meticulously planned clinical trials is crucial for translating these insights into effective treatments for human diseases, ensuring thorough assessment and validation across diverse patient populations.

### 4.1 Target Discovery

Cancer immunotherapy using ICIs such as PD‐1/PD‐L1 inhibitors has been well established for various types of diseases. Microbiota can affect the efficacy of immunotherapy, mainly acting as inhibitors at immune checkpoints [143]. Most research concerning the target usually focuses on ICs. A study demonstrated that *Bifidobacterium infantis* can modulate the PD‐1 signaling pathway and immune responses in mice with IBD, thereby exerting immunoprotective effects akin to ICIs. Specifically, this bacterium has been shown to enhance the proliferation of CD4^+^, CD25^+^, and Foxp3^+^ Tregs in both the spleen and peripheral blood. Moreover, it promotes the expression of Foxp3 within the intestinal tract through activation of the PD‐1/PD‐L1 axis [144]. *Bifidobacterium longum* 420 oral cancer vaccine as an adjunct to ICIs was enhanced in a mouse model of renal cell carcinoma [145]. These findings underscore the pivotal role of the microbiome in modulating the efficacy of PD‐1‐targeted ICIs. By elucidating the interplay between the microbiota and the tumor immune landscape, these studies have laid the groundwork for innovative strategies that harness microbial interventions to enhance ICI efficacy and overcome resistance. Specifically, they highlight the potential of microbiota‐based approaches to augment the antitumor effects of PD‐1 inhibitors, thereby expanding the therapeutic arsenal for cancer treatment.

Other discoveries of new targets are emerging. Microbiome‐derived metabolite TMAO drove antitumor immunity [146]. It stimulated immune activation and enhanced responses to immune checkpoint inhibitors in pancreatic cancer. A study demonstrated that microbiota‐derived acetate boosted host immunity through the NLRP3 pathway [81]. Microbiota could regulate postprandial GLP‐1 responses via ileal BA–TGR5 signaling. Consequently, this signaling pathway may represent a novel therapeutic target for diabetes treatment [147]. Microbiota regulates radiotherapy‐associated antitumor immune responses toward hepatocellular carcinoma through STING signaling. This enhances overall immunity [148]. Researchers identified new signatures for detecting the acute deterioration of UC through targeting microbiota and BAs metabolism. They demonstrate the preventive and therapeutic potential of a microbiota‐derived metabolite, 12‐KLCA [149]. Specifically, 12‐KLCA inhibited IL‐17A secretion from colonic group 3 innate lymphoid cells, thereby preventing acute flare‐ups of UC.

### 4.2 Organoid Screening

The challenges of deducing causation from metagenomic microbiome sequencing studies, as well as the differences between mice and humans, have spurred the progression of advanced in vitro models to simulate the interactions between microbiota and immunity.

Microbiota‐derived butyrate could restrain tuft cell differentiation by HDAC3, and thus regulate intestinal type 2 immunity [150]. Specially, colonization with butyrate‐producing bacteria restricted the differentiation of stem cells and inhibited HDAC3 in human intestinal organoids, effectively blocking tuft cell expansion. Butyrate could also attenuate the activation of guanylin cyclase C‐induced pathways by linaclotide in patient‐derived intestinal organoids [151]. While butyrate has been widely studied, acetate also holds promise as it appears to be nontoxic to epithelial cells. A study demonstrated that high concentrations of acetate could protect the intestinal barrier and exert anti‐inflammatory potentials in organoid‐derived epithelial monolayers from patients with UC [152]. In a coculture system involving ileum organoids and SC06, SC06 repaired LPS‐induced organoid damage by activating the AhR/STAT3 pathway, and enhanced intestinal stem cells [153]. Researchers validated that *Lactobacillus plantarum* alleviated NASH‐related inflammation by enhancing L‐arginine production in liver organoids [154]. Another study utilized organoids derived from CRC patients to assess the impact of microbiota on intestinal tumorigenesis. They revealed that microbial‐derived N‐acetylmuramic acid mitigated CRC through the AKT1 pathway [155]. Adult stem cell‐derived organoids could be employed to investigate the early stages of mycobacterial infection in humans, thereby offering new avenues for fundamental and therapeutic tuberculosis research [156]. The bacterial protease high temperature requirement A (HtrA) is a key factor triggering the multistep pathogenesis of *H. pylori* in organoid‐based epithelial models. HtrA‐dependent E‐cadherin shedding ruined gut barrier function in gastric organoids [157]. The postbiotic gliadin peptide P31‐43 from *Lactobacillus paracasei* CBA L74 induced mTOR/NFκB activation and reduced autophagy in intestinal organoids derived from celiac disease patients [158].

### 4.3 Animal Validation

Numerous preclinical animal experiments have shown how the gut microbiota can influence immune responses and contribute to disease development. These animal studies have provided a foundation for understanding the mechanisms underlying the microbiota and diseases.

*A. muciniphila* mitigated colonic damage in mice with DSS‐induced acute colitis by inhibiting the proinflammatory phenotype switching of Macs through the HDAC5/DAB2 signaling axis [159]. In a recent study, *A. muciniphila* was found to enhance the synthesis of retinoic acid in DCs, thereby modulating the activity of IL‐22 and effectively mitigating colitis in mice [160]. Firmicutes also play a crucial role in colitis. The probiotic strain *B. licheniformis* ZW3 was found to mitigate DSS‐induced colitis and promote intestinal homeostasis [161]. *C. butyricum* and the extracellular vesicles it produces have been shown to regulate gut homeostasis and significantly ameliorate acute experimental colitis [162]. The administration of *B. coagulans* BACO‐17 has been shown to inhibit and potentially reverse the progression of RA, thereby significantly ameliorating its symptoms [163]. Bacteroidota repressed the clearance of amyloid‐beta, promoting plaque deposition in Alzheimer's disease (AD) mouse models [164]. Mice receiving *Bacteroides plebeius* orally demonstrated a distinct gut microbiota composition, and colonization of *B. plebeius* inhibited the colon tumor development triggered by DSS [165]. *Bifidobacterium bifidum* demonstrated its effects of antitumor effects in human gastric cancer xenograft models [166]. *A. muciniphila* prevented cold‐related atrial fibrillation in rats by modulation of TMAO‐induced cardiac pyroptosis [167]. Researchers compared the efficacy of *P. histicola* MCI 001 with a tumor necrosis factor inhibitor in treating arthritis. It indicated that *P. histicola* MCI 001 can serve as a therapeutic agent, as it is capable of suppressing rheumatoid factor levels and antigen‐specific humoral responses [168]. Another experimental study revealed that *Parabacteroides goldsteinii* JCM 13446 administered orally, could reach the colon, cross the intestinal barrier, and travel to the inflamed skin in psoriasis‐like mice, leading to suppressed epidermal hyperplasia, decreased infiltration of inflammatory cells into skin lesions, and significant improvement in both local skin and systemic inflammation [169]. *L. plantarum* DP189 mitigated α‐synuclein aggregation in MPTP‐induced PD mice by regulating oxidative damage, inflammation, and microbiota imbalances [170].

Collectively, current studies indicate that microbiota holds promise as a therapeutic target for many underlying diseases (Table 1). However, additional clinical trials are required to establish its safety and efficacy.

**TABLE 1:** Summary of animal validation.

Bacteria	Subject	Disease	Molecular mechanism	Major findings
*A. muciniphila*	C57BL/6 mice	Colitis	HDAC5/DAB2 axis	↑Disease activity index; ↓weight loss, inflammatory injury [159]
*A. muciniphila*	C57BL/6J mice	Colitis	JAK2–STAT3 signaling pathway	↑IL‐22 [160]
*B. licheniformis ZW3*	C57BL/6 mice	Colitis	Aminoacyl‐tRNA biosynthesis	↑Inflammatory markers IL‐1β and IL‐6; ↓Escherichia‐Shigella [161]
*C. butyricum*	C57BL6J mice	Colitis	FoxO signaling pathway	↑LPS, IL‐6, TNF‐α; ↓inflammatory infiltration, epithelial barrier disruption, and crypt destruction [162]
*B. coagulans BACO‐17*	BALBc mice	RA	IL17 pathway	↑Articular cartilage and synovium [163]
*B. fragilis ATCC 25285*	APP/PS1‐21 Jucker C57BL/6J mice	AD	Microglia phagocytic function	↓Aβ clearance; ↑amyloid plaques [164]
*B. plebeius JCM 12973/DSM17135*	C57BL/6JN mice	Colon cancer	Beneficial metabolites	↓Colon tumor development [165]
*A. muciniphila*	SD rats	Atrial fibrillation	TMAO modulation	↑M1 Macs infiltration; Casp1‐p20 and cleaved‐GSDMD expression [167]
*P. histicola MCI 001*	DQ8 mice	Arthritis	Immune modulatory effects	↓Rheumatoid factor and antigen‐specific humoral response [168]
*P. goldsteinii JCM 13446*	BALB/c mice	Psoriasis	Immune modulatory effects	↓Skin and systemic inflammation [169]
*L. plantarum* DP189	C57BL/6 mice	PD	Nrf2/ARE and PGC‐1α pathways	↑SOD, GSH‐Px, and IL‐10; ↓α‐SYN accumulation [170]

*Abbreviations*: AD, Alzheimer's disease; PD, Parkinson's disease; RA, rheumatoid arthritis.

### 4.4 Clinical Trial Design

Many clinical trials have delved into the connection between microbiota and diseases. These trials shed light on how changes in microbiota can sway immune responses and play a role in disease onset.

Vaginal candidiasis (VC) has changed the microbiota of vaginal regions and gut microbiota profiles, alleviating the gut microbiota that are crucial for gut nutrient availability, protection, and immunity [171]. Administrating Lactobacilli probiotics has prevented such a shift, leading to better modulated gut and vaginal microenvironment during VC. For patients with psoriasis, a combination of antipsoriatic local therapy and probiotic supplementation brought improved disease activity measures. This included reduced inflammatory markers and skin thickness compared with those who did not receive supplementation [172]. Moreover, among the 15 out of 42 patients in the intervention group who underwent gut microbiota analysis, a favorable alteration toward an anti‐inflammatory profile was observed after 12 weeks of probiotic and prebiotic supplementation. Pathogenic mechanisms underlying distal symmetric polyneuropathy (DSPN), a common complication in patients with diabetes, have been explored. In a randomized, double‐blind, placebo‐controlled trial, 22 patients who received fecal microbiota transplants from healthy donors experienced significant amelioration of DSPN symptoms, independent of glycemic control, compared with the 10 patients who received a placebo [173]. Adjuvant probiotics, including *Lactobacillus salivarius* subsp. salicinius AP‐32, *Bifidobacterium animalis* subsp. lactis CP‐9, and *Lactobacillus johnsonii* MH‐68 demonstrated reducing inflammatory cytokines and glycemic levels in patients with T1D [174]. Among adults with laboratory‐confirmed *Clostridium difficile* infection (CDI) who were related to CDI at high risk for recurrence, high‐dose VE303, namely, the combination of several kinds of probiotics, significantly prevented recurrent CDI compared with placebo [175]. *Lacticaseibacillus rhamnosus* GG has demonstrated its potential as an adjunctive therapy in pediatric atopic dermatitis. The beneficial effects on disease severity and quality of life were accompanied by regulating the gut and skin microbiome [176]. Supplementation with *Lactobacillus acidophilus* LB along with atomoxetine for 3 months had a beneficial effect on attention‐deficit/hyperactivity disorder (ADHD) symptomology and cognitivity [177]. This suggested that probiotics may be a promising adjunctive treatment for managing ADHD. Compared with placebo, *L. paracasei* supplementation led to a decrease in remnant cholesterol, which was significantly related to improved endothelial function. In subjects who strictly adhered to the trial protocol, *L. paracasei* treatment also reduced triglycerides, alleviated the severity of metabolic syndrome (Mets), and delayed weight gain [178]. Daily oral administration of *L. rhamnosus GG* was significantly associated with ameliorated liver injury [179]. Group B Streptococcus (GBS) is a main cause of bacterial neonatal sepsis. Oral administration of *Ligilactobacillus salivarius* V4II‐90 was a safe and effective tactic to reduce GBS colonization rates at the end of pregnancy, thus alleviating the need for intrapartum antibiotic prophylaxis for both mothers and their infants [180].

Collectively, these clinical findings underscore the complex interplay between microbiota and the immune system, highlighting the need for further investigation into the mechanisms by which microbiota contribute to immune‐mediated diseases (Table 2).

**TABLE 2:** Summary of representative clinical trials.

Bacteria	Subject	Disease	Identifier	Major findings
Lactobacillus probiotics	Pregnant women with VC	VC	NCT03940612	↓Lessening of gut microbiota [171]
Microbiota from DSPN patients	Patients received fecal microbiota transplants from healthy donors	DSPN	ChiCTR1800017257	↓DSPN [173]
Lactobacillus and Bifidobacterium probiotics	T1D patients between 6 and 18 years of age	T1D	NCT03880760	↑ Populations of beneficial microbiotas in the gut of patients with T1D received probiotics treatment [174]
Clostridia probiotics (VE303)	Adults at high risk for CDI recurrence	CDI	NCT03788434	High‐dose VE303 prevented recurrent CDI compared with placebo [175]
*L. rhamnosus* GG	Patients aged 6–36 months	Atopic dermatitis	NCT03863418	↑Gut and skin microbiome [176]
*L.acidophilus* LB	ADHD patients	ADHD	NCT04167995	↑Focus attention [177]
*L. paracasei* 8700	Mets patients	Mets	NCT05005754	↓Triglycerides, MetS severity [178]
*L. rhamnosus* GG	Individuals with alcohol use disorder and moderate alcohol‐associated hepatitis	Hepatitis	NCT01922895	Liver injury [179]
*L. salivarius* V4II‐90	Pregnant participants positive for vaginal–rectal colonisation	Neonatal sepsis	NCT03669094	The rates of GBS colonisation at the end of pregnancy [180]

*Abbreviations*: ADHD, attention‐deficit/hyperactivity disorder; CDI, Clostridioides difficile infection; DSPN, distal symmetric polyneuropathy; FMT, fecal microbiota transplants; GBS, group B Streptococcus; Mets, metabolic syndrome; T1D, type 1 diabetes; VC, vaginal candidiasis.

Emerging technologies are expected to enhance precision These developments highlight the potential of harnessing the microbiota to restore immune balance and improve human health Future research must prioritize mechanistic studies, long‐term safety assessments, and inclusive trials that cover interindividual variability.

## 5 Treatment Strategies in the Microbiota–Immune System

**FIGURE 4:**
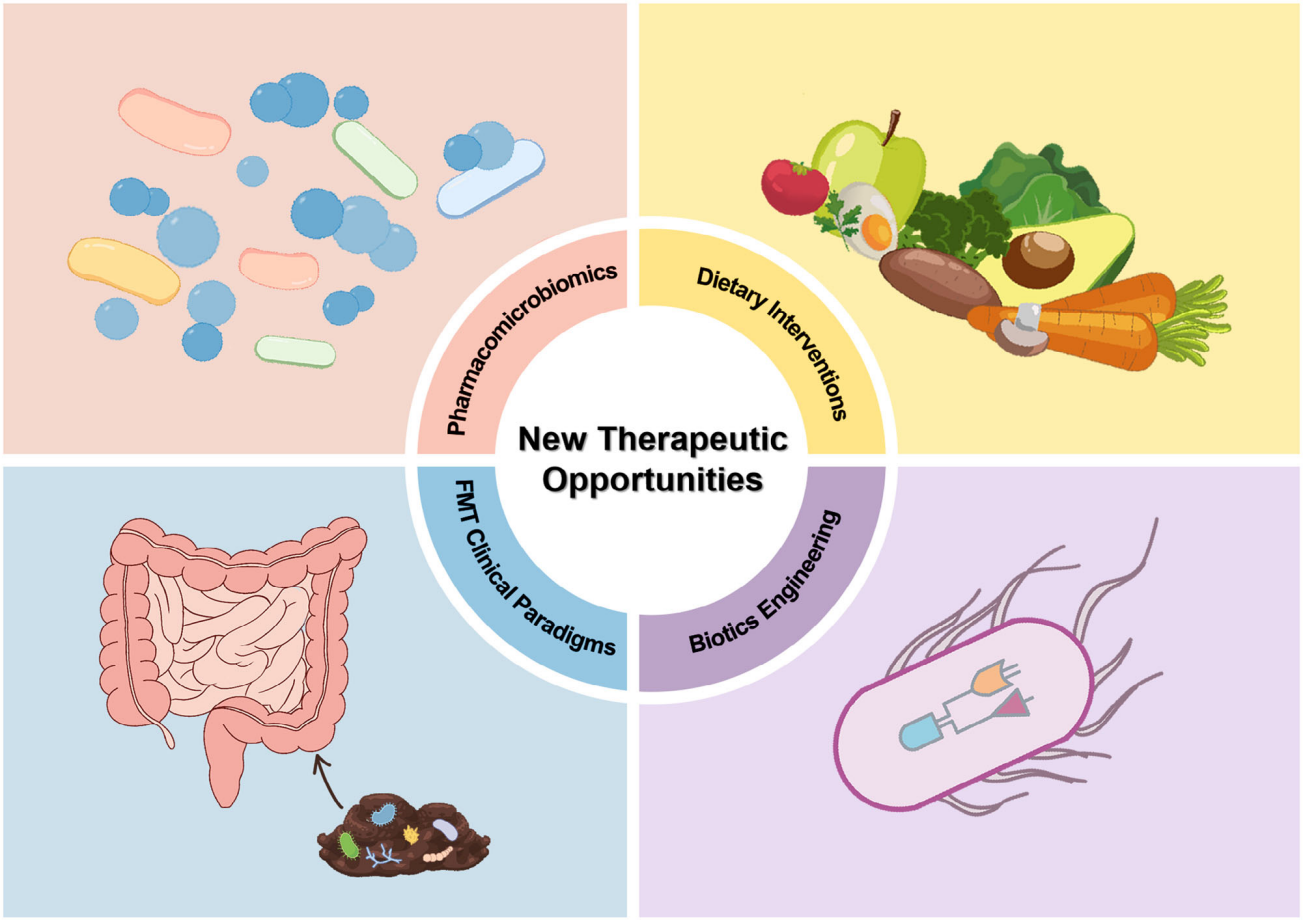
New therapeutic opportunities in the microbiota–immune system. Four key methods, dietary interventions, biotics engineering, FMT clinical paradigms, and pharmacomicrobiomics provide new opportunities in the microbiota–immune system. *Abbreviation*: FMT, fecal microbiota transplantation.

The microbiota is now considered a promising strategy for drug design and personalized therapy. Diet and host genetics regulate drug efficacy, pharmacomicrobiomics focuses on the alterations linked with the microbiome that modulate drug response. Up to now, some advancements have been achieved in the field of microbiome therapy. The following will introduce four key methods: dietary interventions, biotics engineering, FMT clinical paradigms, and pharmacomicrobiomics, focusing on their mechanisms, clinical applications, and the latest developments (Figure 4). With the advances in the microbiome, therapy and pharmacomicrobiomics‐associated technologies will provide novel insights into the interactions between drugs and the microbiome.

### 5.1 Dietary Interventions

Dietary components can influence changes in microbiota, and in return, diet also affects the production of microbiota‐derived metabolites, such as SCFAs, Trp, and BAs.

Diet has a significant impact on the composition and function of the microbiota. Dietary interventions rich in dietary fiber and polyphenols, such as the Mediterranean diet, could increase the abundance of beneficial bacteria (such as *Faecalibacterium* and *Roseburia*), exert anti‐inflammatory effects by enhancing the production of SCFAs and reducing the levels of proinflammatory metabolites [181]. In a collagen‐induced arthritis mouse model, *P. copri* exacerbated arthritis, and a HFD further amplified this effect. Studies have found that *P. copri* can efficiently degrade complex fibers, producing proinflammatory metabolites such as succinate and fumarate, thereby promoting inflammatory responses and worsening RA symptoms. These findings emphasized the importance of considering microbiota composition when evaluating the pathological effects of dietary interventions on RA, and indicated that an HFD may not be universally beneficial for RA patients, especially those with specific microbial imbalances [32]. Fermented foods like kimchi and kefir may help reduce asthma symptoms. They contain lots of probiotics and bioactive compounds. These can adjust the immune system and cut down inflammation. Studies show that eating such foods might cut down asthma attacks. They can trigger anti‐inflammatory cytokines like IL‐12 and change the microbiota. This affects the gut‐lung axis [182].

### 5.2 Biotics Engineering

Bacterial engineering employs genetic tools to modify the genomes of bacteria, enabling them to produce modified proteins, peptides, nucleic acids, and other biomolecules for therapeutic applications [183]. These engineered bacteria can target diseased tissues or organs, detect specific biomarkers in the disease microenvironment, and induce desired physiological changes. Intracellular metabolic pathways can be precisely designed to regulate gene expression, synthesize biologically active molecules, and deliver therapeutic payloads to affected tissues [184].

Among the most extensively studied engineered bacterial species are *L. lactis*, *S. typhi*, and *E. coli* Nissle, which have been utilized as drug carriers in vaccines for diseases [185]. The engineered probiotic *E. coli* Nissle 1917, specifically tailored for tumor targeting, exerts a potent inhibitory effect on colorectal tumorigenesis and concurrently modulates microbiota homeostasis in mice [186]. In a study addressing IBD induced by DSS, engineered E. coli Nissle 1917 expressing IL‐2 demonstrated significant therapeutic potential. Specifically, this engineered strain effectively activated Tregs, which in turn modulated innate immune responses and restored microbiota homeostasis [187]. These actions collectively alleviated inflammation and facilitated the repair of the colonic epithelial barrier. Engineered *E. coli Nissle* expressing IL‐34 has been shown to directly target damaged intestinal mucosa, upregulate IL‐34 expression, and enhance the expression of tight junction proteins in IECs, thereby alleviating experimental colitis in mice [188]. Engineered bacteria delivering the cytokine offering a novel strategy for UC treatment, with interleukin may serve as a therapeutic target. Using CRISPR/Cas9 technology, researchers achieved the stable expression of the HIV‐1 membrane‐proximal external region extended epitope on the surface of recombinant probiotic *E. coli Nissle 1917* [189]. This innovative approach leverages the probiotic properties of microbiota to serve as a novel platform for displaying HIV‐1 antigens, potentially enhancing immune recognition and response. Genetically engineered *L. lactis* carrying hCAP18 cDNA effectively mitigates DNBS‐induced colitis in C57BL/6 mice by enhancing the expression of IL‐17A and IL‐10 cytokines [190].

While engineered bacteria hold great potential, safety concerns remain. Gene leakage and potential allergic reactions in individuals with weakened immune systems are significant issues. Future research must focus on selecting appropriate genetic tools and designing precise molecular switches to control and monitor the effects of these bacteria. Additionally, a comprehensive understanding of the synthetic biology, heterologous expression, and secreted substances of the selected bacteria, along with strict regulation of bacterial dosage, is essential to balance therapeutic efficacy and bacterial toxicity.

### 5.3 FMT Clinical Paradigms

FMT is to move fecal microbiota from healthy people to patients. It aims to bring back microbial diversity in recipients. The United States Food and Drug Administration has approved it for treating recurrent CDI. However, it is now also being explored for the treatment of immune‐related diseases. This is because FMT can restore the balance of the microbiota, which is crucial for immune system regulation, by modulating immune responses through changing the composition of the microbiota [191].

Research has demonstrated that FMT enhances the efficacy of anti‐PD‐1 inhibitors in patients with unresectable or metastatic solid tumors refractory to anti‐PD‐1 therapy [192]. Another research study revealed FMT notably ameliorated insulin resistance, reduced body mass index, and altered gut microbial communities in T2D patients, primarily through donor‐derived microbiota colonization [193]. In another double‐blind, randomized controlled trial, FMT also showed its positive effects in T2D [194]. Repeated FMTs increased the duration and level of microbiota engraftment in obese individuals with T2D. Furthermore, combining FMT with lifestyle intervention led to more favorable alterations in patient microbiota and improvement in liver stiffness and lipid profile. Multidonor FMT, combined with an anti‐inflammatory diet, significantly generated sustained deep remission in patients with mild‐to‐moderate UC [195]. FMT could improve the therapeutic outcomes in patients with NAFLD, with higher clinical efficacy demonstrated in lean NAFLD patients compared with obese NAFLD patients [196]. The first clinical trial concerning FMT in active SLE patients provided solid evidence that FMT may be a safe, feasible, and promising treatment option for SLE patients, primarily by modifying the microbiota and its metabolic profile [197].

Modulating the microbiome through FMT can potentially enhance the therapies of microbiota. Therefore, more clinical trials need to further investigate the application of FMT to solve health problems.

### 5.4 Pharmacomicrobiomics

The microbiota can metabolize and biotransform a broad range of xenobiotic compounds and drugs. It also can modify the toxicity and activity of these substances. Thus, it impacts how a host responds to xenobiotics and drugs. This emerging area of study is known as pharmacomicrobiomics. Pharmacomicrobiomics is now emerging as a vital vehicle in the microbiota–immune system.

The immune regulatory effects of probiotics have been widely studied. In allergic diseases, probiotics can reduce IgE levels and mast cell activation. For example, a randomized controlled trial showed that perinatal supplementation with *L. rhamnosus* could reduce the incidence of childhood asthma by modulating the Th1/Th2 balance [198]. Mice administered with *P. goldsteinii* exhibited decreased aspirin‐induced damage to the intestinal niche and gut barrier [36]. Emerging evidence also supports the use of probiotics in the management of psoriasis by inhibiting the IL‐17/IL‐23 pathway, thereby reducing the severity of psoriatic plaques [199]. However, strain‐specific effects require personalized approaches to optimize efficacy.

Prebiotics, as nondigestible fibers, have become a promising strategy for enhancing beneficial microbiota and regulating gut health, especially in the context of diseases. These fibers can selectively stimulate the growth of beneficial bacteria, particularly those that produce SCFAs and thus maintaining intestinal barrier function and reducing inflammation by inhibiting the NF‐κB signaling pathway [200]. Prebiotics have shown potential applications in the treatment of various immunological diseases, with main mechanisms including modulating the microbiota structure, influencing immune system function, and reducing the production of inflammatory mediators. However, current research results still have certain limitations and require further clinical trials to verify their efficacy and safety [201].

Methods targeting pharmacomicrobiomics have the potential to revolutionize the treatment of diseases, but still face challenges. Probiotics, due to their strain‐specific effects, require further optimization. New therapeutic treatment needs to be found in the microbiota–immune system.

## 6 Conclusion and Prospects

The microbiota and immunity represent a fascinating and rapidly evolving area of research. The past research has witnessed significant advancements in the understanding of how different phyla of the microbiota interact with the immune system at molecular and functional levels. However, there is a big gap between lab discoveries and real treatments. Without more clinical trials, we are stuck spinning our wheels.

This microbiota–immune system is incredibly complex. To crack the code, we need to pinpoint exactly which molecular pathways and chemical signals matter most. Cutting‐edge tools like single‐cell analysis and advanced gene sequencing could help us map these intricate relationships at unprecedented resolution. And let us not overlook the potential of personalized treatments—imagine therapies customized to a patient's unique microbial makeup.

Understanding this hidden partnership between our microbes and immune system could revolutionize healthcare. But getting there requires working together. As we push forward, we must tackle tough questions about how exactly our microscopic residents influence different diseases. Crack those mysteries, and we open doors to precision treatments that tweak this delicate balance for better health.

## Author Contributions

J.J.Z. wrote the original draft and finished the figures and tables. Z.M.H., G.Q.W., and Y.X.M. organized the figures and tables, edited the manuscript, and provided supervision. F.Z. conscientiously wrote, revised, and checked the whole review. All authors have read and approved the final manuscript

## Conflicts of Interest

The authors declared no conflicts of interest.

## Ethics Statement

The authors have nothing to report.

## Data Availability

The authors have nothing to report.
